# Molecularly Imprinted Polymer-Based Sensors for Protein Detection

**DOI:** 10.3390/polym15030629

**Published:** 2023-01-26

**Authors:** Semra Akgönüllü, Seçkin Kılıç, Cem Esen, Adil Denizli

**Affiliations:** 1 Department of Chemistry, Faculty of Science, Hacettepe University, 06800 Ankara, Turkey; 2 Department of Chemistry, Faculty of Science, Aydın Adnan Menderes University, 09010 Aydın, Turkey

**Keywords:** molecularly imprinted polymers, protein detection, molecular imprinting, sensors, biomolecules, nanofilm

## Abstract

The accurate detection of biological substances such as proteins has always been a hot topic in scientific research. Biomimetic sensors seek to imitate sensitive and selective mechanisms of biological systems and integrate these traits into applicable sensing platforms. Molecular imprinting technology has been extensively practiced in many domains, where it can produce various molecular recognition materials with specific recognition capabilities. Molecularly imprinted polymers (MIPs), dubbed plastic antibodies, are artificial receptors with high-affinity binding sites for a particular molecule or compound. MIPs for protein recognition are expected to have high affinity via numerous interactions between polymer matrices and multiple functional groups of the target protein. This critical review briefly describes recent advances in the synthesis, characterization, and application of MIP-based sensor platforms used to detect proteins.

## 1. Introduction

Molecular imprinting is a well-established technique used to obtain synthetic materials with “molecular memory”. Simply, it is the process of performing the polymerization of proper functional monomers in the presence of a related target molecule called a “template” [1,2]. Molecular imprinting is an outstanding technology that incorporates molecular affinity sites into homogeneous polymeric matrices. Using this technique, selective polymeric matrices have been successfully prepared for a variety of templates—from viruses to biomacromolecules—regardless of size [3]. Specific molecular recognition is the essential process controlling both biological form and function. This fascinating and ubiquitous biological phenomenon is mainly mediated by proteins. Proteins are complex assemblies of macromolecules with regions that can interact and bind controllably and specifically with target molecules [3]. Moreover, proteins constitute one of the most significant categories of biomarkers [4]. They have broad applicability in numerous fields, including the selective detection of protein targets, clinical diagnostics and recuperative monitoring, the control of bioreactors, and the detection of organisms and toxins, along with bioterror agents.

Some challenges can be raised when a template is a biomacromolecular protein, as classical bulk processes developed for small analytes often need to address the features of protein targets. These challenges mainly arise due to the actual features of proteins: (i) because of their fragility, potentially irreversible conformational changes can take place during the polymerization process [5], and the rebinding of the natural conformation to such imprinted sites is not preferred; (ii) moreover, the macro-size of proteins beclouds their separation from or rebinding to a highly cross-linked three-dimensional polymeric structure generally employed in small-analyte imprinting, means that biomacromolecules can be permanently captured in the polymeric substance during the imprinting process (iii) the potential binding sites on the surfaces of proteins may cause cross-reactivity of the imprinted polymers and non-specific adsorption onto a bulk polymeric substance [1]. Furthermore, the span of protein targets applied is slim and limited to templates with features that make the imprinting process easier. Accordingly, proteins incorporating satisfactory conformational stability and various physical–chemical characteristics are generally preferred. Such properties include high isoelectric points and glycosylation. They facilitate the formation of potent and selective interactions, or the mixture of the two, namely, the electrostatic interactions between positively charged proteins and negatively charged polymers, glycan fragments, and (aminophenyl)boronic acid monomers. Such formation strongly indicates that enhancements in selectivity and affinity are necessary in order to consider macromolecular imprinting other than as a proof-of-concept level technique.

MIPs are analogues of natural antibody–antigen and enzyme–substrate systems. Therefore, the “key-lock” mechanism is mimicked during the synthesis phase to recognize the target molecule selectively. Compared to natural antibodies, MIPs display many attractive characteristics, such as simple preparation, cost-effectiveness, stability, reusability, and tolerance to harsh conditions including pH, high temperature, organic solvents, etc. Owing to these superior features, MIPs have demonstrated remarkable potential in many application areas, including separation [6], bioassays [7], disease diagnosis [8], cancer therapy [9], single-cell analysis [10], drug delivery [11], bioimaging [12], toxin neutralization [13], and so on. Herein, a crucial examination of protein imprinting and MIP-based sensor development methods will be presented, with the objective of identifying the most promising approaches to the development of an MIP-based sensor with sensitive and selective properties for a protein target. The overhead scheme reflecting the outline of this review is as follows: the description of MIPs (Section 2) and a general overview of the MIP procedure (Section 2.1 and Section 2.2) are presented, followed by a broad review of the sensor modifications, applications, and performance of MIP-based sensors (Section 3) with respect to protein detection. Subsequently, this review’s critical conclusions are presented (Section 4).

## 2. Molecularly Imprinted Polymers (MIPs)

MIPs are functional porous materials that offer analyte-based targeting via high-affinity binding sites customizable to their size, shape, and functionality [14,15]. Molecular imprinting was initially developed by Polyakov [16] in the 1930s. Advancing to the 1970s, Wulff and Sarhan [17] made further advancements; however, in the 1990s, Mosbach and co-workers [18] introduced the non-covalent imprinting approach, which has augmented the popularity of MIPs. It is based on a templating technique through which specific molecular recognition sites in networks of synthetic porous polymers can be produced [19].

### 2.1. MIP Design

**Figure 1:**
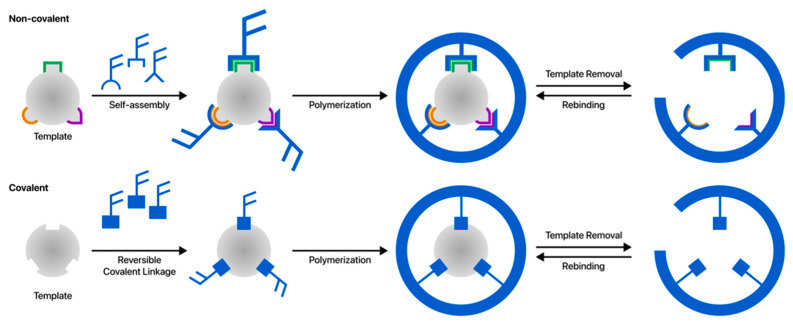
MIP procedures: non-covalent and covalent imprinting. (Adapted from [29]).

Polymeric receptors such as MIPs are crucial to multi-synthetic processes because of their recognition properties [20]. The molecular imprinting technique involves the formation of a pre-polymerization complex of the target molecule acting as a template with functional monomers capable of undergoing weak non-covalent interactions [21] or forming reversible covalent bonds [22] with the target, as shown in Figure 1. The polymeric matrix is synthesized in the presence of the template molecule. The next step of the process is that the functional groups are kept in place by a highly cross-linked polymer matrix, thus trapping the template in a rigid cavity. Consequently, the pre-polymerization complex thus formed is polymerized [23]. As the polymerization process ends, the template is removed from the resulting polymer matrix to obtain MIP. This process results in polymeric materials containing micro- or nano-sized cavities that match the functionality, shape, and size of the template molecule. These cavities are used for the selective binding of target species structurally and chemically similar to the original template analyte [24,25,26,27]. Numerous methods have been developed to prepare MIPs, which are categorized as covalent, non-covalent, semi-covalent, and metal–ion exchange imprinting methods. However, the non-covalent approach has been the most widely used among these strategies [28].

Although molecularly imprinted polymers are prepared using different methods, such as bulk imprinting, surface imprinting, and epitope imprinting, all methods use the same fundamental principles:

(i) A pre-polymerization complex is formed, incorporating the template molecule covalently or non-covalently linked to functional monomer(s).
(ii) The polymer is crafted using an initiator with a pre-polymerization complex and crosslinker.
(iii) The template is removed from the polymer, forming specific binding sites specific to the template molecule. Therefore, MIP cavities are suitable for selective template rebinding.
(iv) When the MIP interacts with the template-containing sample in the complex medium, only the template molecule binds selectively/specifically to the cavities.

#### MIP Preparation Process

Usually, one to four monomers are used to prepare MIPs. The use of a proper preparation method is crucial to produce MIPs with desired properties. In general, free radical polymerization and sol–gel processes are fundamental to MIP preparation mechanisms. There are various molecular imprinting techniques [30]:

*Bulk imprinting:* In the bulk-imprinting technique, a target analyte is incorporated directly into the polymer matrix a whole [31]. Grinding and sieving should be performed for further MIP application.

*Surface imprinting:* In the surface-imprinting technique, binding sites are formed on the surface of the polymer matrix [32].

*Epitope imprinting:* In epitope imprinting, a pseudo template based on the partial structure or fragment of the target protein is used to prepare imprinted polymer materials [33].

*Nanoimprinting:* Recently, the development of molecular imprinting nanotechnologies has gained significant interest, particularly with respect to nanostructured MIPs (N-MIPs), which show greatly enhanced properties in contrast to bulk MIPs. This method provides good accessibility to target species and improves binding kinetics and capacity [30].

A template molecule, a functional monomer, a crosslinker, a polymerization initiator, and a solvent (porogen) are fundamental elements of a typical MIP synthesis procedure. Since many factors are customizable and can severely impact the result of any polymerization reaction, a possible way to create superior MIPs lies in the type and amount of monomers, crosslinkers, and initiators, also important parameters are the type/amount of solvent; the temperature; and the type and duration of the reaction, which has already been performed many times. Template molecules, functional monomers, and crosslinkers, hence the term “the three main elements of molecular imprinting,” need to be specifically investigated [30].

MIPs can bind to the target molecule selectively or specifically, with high-affinity binding occurring whether the target molecule is in a complex matrix such as a biological fluid [34], on a cell’s surface, or in tissue [35]. Functional monomers provide a hydrogen-bonding function or a covalent-bonding reactive substituent with the target. Examples of functional monomers are acrylamide, methyl methacrylate (MMA), methacrylic acid (MAA), aniline, and pyrrole. Following the utilization of a suitable crosslinker (e.g., ethylene glycol dimethacrylate (EGDMA)) and a polymerization initiator, usually azobis(isobutyronitrile) (AIBN), polymerization is initiated by heating or UV radiation [36]. Today, MIPs can be prepared in such varieties as macro, micro, and nanomaterials [37] thanks to the technical advances in the specialization of polymer synthesis [38].

Comparatively, MIPs uniquely and mainly offer structure predictability, recognition specificity, and applicational universality. As a result, they have received overall engagement due to their high stability, simple preparation, excellent robustness, and low cost. This level of engagement allows them to be exploited in many fields, such as purification and separation, chemical- or bio-sensing, artificial antibodies, drug delivery, catalysis, and degradation [30]. The mechanical, chemical, and thermal stability; ease of preparation; and relatively low cost of MIPs compared to biological recognition materials make them essential for different analytical applications [39]. One of the attractive features of the molecular-imprinting technique is that it can be applied to a broad range of target molecules. Such a property supports their use in various application fields, such as drug delivery processes, environmental samples, and preparing food, toxins, pesticides, and other molecules, which are otherwise difficult to isolate, identify, and detect [40]. Over the recent years, the field of molecular imprinting has grown quickly and expanded tremendously. Furthermore, currently, there are completely novel areas where imprinted polymers are being used [41]. MIPs can theoretically be prepared for any molecule or compound. However, the best results have been obtained for substances with molecular weights between 200 and 1200 Da [42]. Although MIPs with major selectivity for low molecular weight molecules are being synthesized, imprinting with large macromolecules continues to be challenging [41]. Larger templates are less rigid and, therefore, may not facilitate the creation of well-defined binding sites during the imprinting process. Furthermore, the secondary and tertiary structures of macro biomolecules such as proteins can be affected when they are subjected to the thermal or photolytic treatments involved in the synthesis of MIPs. Rebinding is also more difficult, as large molecules such as peptides and proteins cannot easily diffuse into the polymer network to reoccupy binding cavities [43]. The epitope-imprinting technique offers some important advantages such as low cost, easy availability for large template molecules, and high effectiveness with respect to template immobilization on a substrate’s surface [44].

As a synthetic analog, MIPs have been well incorporated into analytical methods, including diagnostics [45], chromatography [46], and sensor platforms [47,48]. MIPs can be prepared on sensor substrates in various physical forms such as thin films, porous microspheres, nanospheres, nanowires, nanostructured films, nanocomposites, and semi-soluble nanogels [49]. MIPs constitute one of the most interesting forms of surface modification, and can be combined with sensors [50]. Moreover, the integration of these artificial recognition materials with nanostructured materials such as carbon dots, carbon nanotubes, gold nanoparticles, and magnetic nanoparticles for conductivity enhancement and signal amplification has been successfully achieved in recent times [51,52,53,54,55]. MIPs have been shown to be reliable and cost-effective materials offering selective detection in various applications, including clinical diagnosis [43], environmental monitoring [56], food control [57], and homeland security [58]. As a multidisciplinary technology, molecular imprinting is rapidly evolving, with developments in polymer science, nanotechnology, biotechnology, analytical chemistry, environmental science, and other related fields. Concurrently, the synergy between technological development and strategic advancements will create stable improvements in molecular imprinting technology and lead to significant breakthroughs. All these desirable factors have resulted in the urge to employ MIPs as recognition elements in chemical- and biosensors [14]. The critical parameters regarding the application of MIPs to sensing devices involve the imprinting factor (*IF*), coupling capacity, and response time [36].

To determine imprinting efficiency, a non-imprinted polymer (NIP) corresponding to an MIP should also be synthesized as a control polymer in the absence of a template molecule. For this reason, the physical, chemical, and functional characterization of MIP and NIP materials commonly involves a wide range of analytical techniques [59].

The physical characterization techniques to determine the surface and morphological features of MIPs include electrochemiluminescence; differential scanning calorimetry (DSC); atomic force microscopy (AFM); MIP particle size analysis, such as dynamic light scattering (DLS), static light scattering, and laser diffraction; specific surface area and pore size calculation (Brunauer–Emmett–Teller (BET) analysis); Raman spectroscopy; swelling tests; scanning electron microscopy (SEM); thermogravimetric analysis (TGA); transmission electron microscopy (TEM); X-ray photoelectron spectroscopy; X-ray powder diffraction; and zeta potential analysis [60].

On the other hand, the chemical characterization techniques include IR spectroscopy, elemental analysis, and NMR spectroscopy. In addition, NMR, IR, and UV–Vis spectroscopy are employed to monitor functional monomer–template molecule interactions that occur in pre-polymerization complexes [60].

### 2.2. Use of Non-Imprinted Polymers (NIPs) and Determination of Imprinting Factor (IF)

Any MIP-based analytical approach consists of synthesizing an NIP as well. This polymer is produced under the same conditions as the corresponding MIP but without the template. Then, the NIP is processed accordingly to reveal the presence of imprinted regions in the MIP [61]. NIPs serve as a “control” to assess the selectivity of the interactions between synthesized MIPs and the template molecule, which is specific for MIPs but not for NIPs [62]. Compared to NIPs, MIPs have better binding capacity and selectivity [63]. The calculated binding ratio between MIPs and NIPs is called the imprinting factor (*IF*) (Equation (1)):

(1) $$IF=k_{MIP}/k_{NIP}$$

Here, *k_MIP_* and *k_NIP_* represent the binding capacities in a monolayer polymer surface.

### 2.3. Combination with Sensors

Chemical- and biosensors are attracting increasing interest in modern analytical chemistry due to the various approaches and techniques necessitated by new requirements and emerging prospects. The fields of interest range from clinical diagnostics to environmental analysis, food analysis to production monitoring, and the detection of illicit drugs and genotoxicity with the addition of chemical warfare agents [14]. A typical sensor consists of two fundamental functional units: a recognition element ‘receptor’ and a translating physicochemical transducer. The receptor is responsible for the selective recognition of the target analyte, whereas the transducer, which can be electrochemical, optical, or mechanical, is responsible for the conversion of the chemical or biological recognition into a measurable signal [64]. 

**Figure 2:**
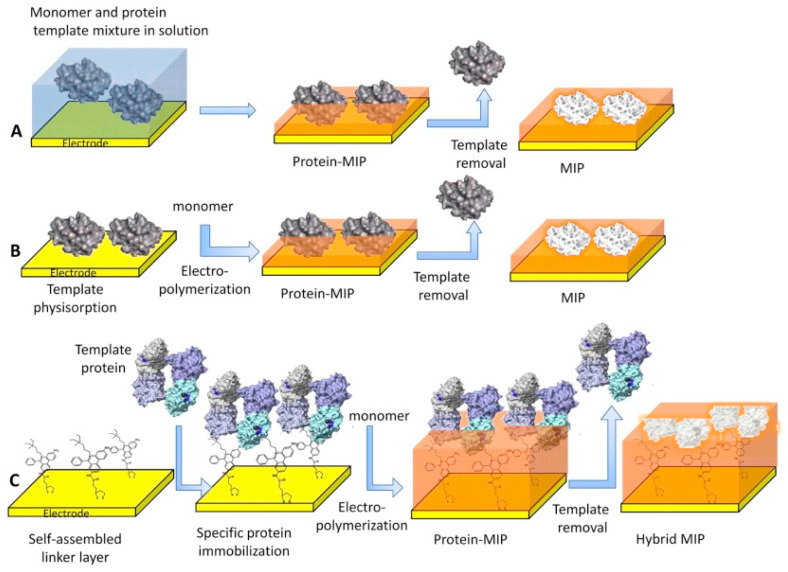
The various surface-imprinting approaches for selective recognition of proteins: (**A**) electropolymerization; (**B**) preconcentration of the protein on the electrode surface; and (**C**) self-assembly on an anchor layer. Reprinted from [1] with permission from Erdőssy et al.

The most known traditional method of producing MIPs uses free radical polymerization; however, for the preparation of molecularly imprinted thin or nano polymer films directly on the electrode’s (or chip’s) surface, electrochemical polymerization is the procedure that is being chosen with greater frequency [65,66,67]. Moreover, MIP NPs (nanoMIPs) have been also synthesized using the solid-phase synthesis technique [68]. The various surface imprinting methodologies used in the synthesis of MIP films for selective recognition of proteins are shown in Figure 2.

## 3. Protein Detection

The most crucial parameters characterizing the performance of MIPs are specific affinity and the high selectivity of protein rebinding, which emphasize the linear range of concentrations such as a low limit of detection (LOD) and cross-reactivity. This review presents MIP sensor applications for protein detection published in the last decade.

### 3.1. MIP-Based Optical Sensors

MIP-based optical sensors are based on the change in the optic features of the device during biorecognition that is to be revealed and measured. There has been a significant increase in the study of MIP-based optical sensors in the literature in recent years [69]. Notably, surface plasmon resonance (SPR) sensor technology has been broadly used for the sensing of various biological and chemical analytes throughout the past decade [70].

#### 3.1.1. Albumin

Human serum albumin (HSA) is the most abundant protein in the human body and contains 585 amino acids at concentrations of almost 3.5–5 g/dL [71]. Albumin protein determination has attracted great interest as a basic and rapid methodology for the early diagnosis of kidney diseases [72]. In a relevant work, Esentürk et al. designed an MIP-based nanoparticle-attached SPR sensor for the detection of microalbumin in a buffer solution and urine sample [73]. The authors synthesized albumin-imprinted nanoparticles using EGDMA and N-methacryloyl-L-leucine methyl ester as a cross-linker and a functional monomer, respectively. The physical characterization of MIP nanoparticles was accomplished using FTIR, DLS, and SEM. A modified SPR sensor chip surface was also examined via contact angle measurement, ellipsometer, and AFM. The LOD and limit of quantification (LOQ) values of the albumin-imprinted SPR sensor were found to be 0.7 pM and 1.9 pM, respectively, within the linear concentration range of 0.15–500 nM. 

Wang and Wei [74] developed an MIP-based SPR sensor chip for the detection of bovine serum albumin (BSA) by the electropolymerization of (3-aminophenyl)boronic acid (3-APBA). The surface morphologies of MIP and NIP films were characterized by SEM analysis and nanosized cavities formed homogeneously on the MIP film surface were observed. Optimization and selectivity studies were conducted for MIP-based SPR sensors and the LOD was found to be 0.02 mg/mL. 

**Figure 3:**
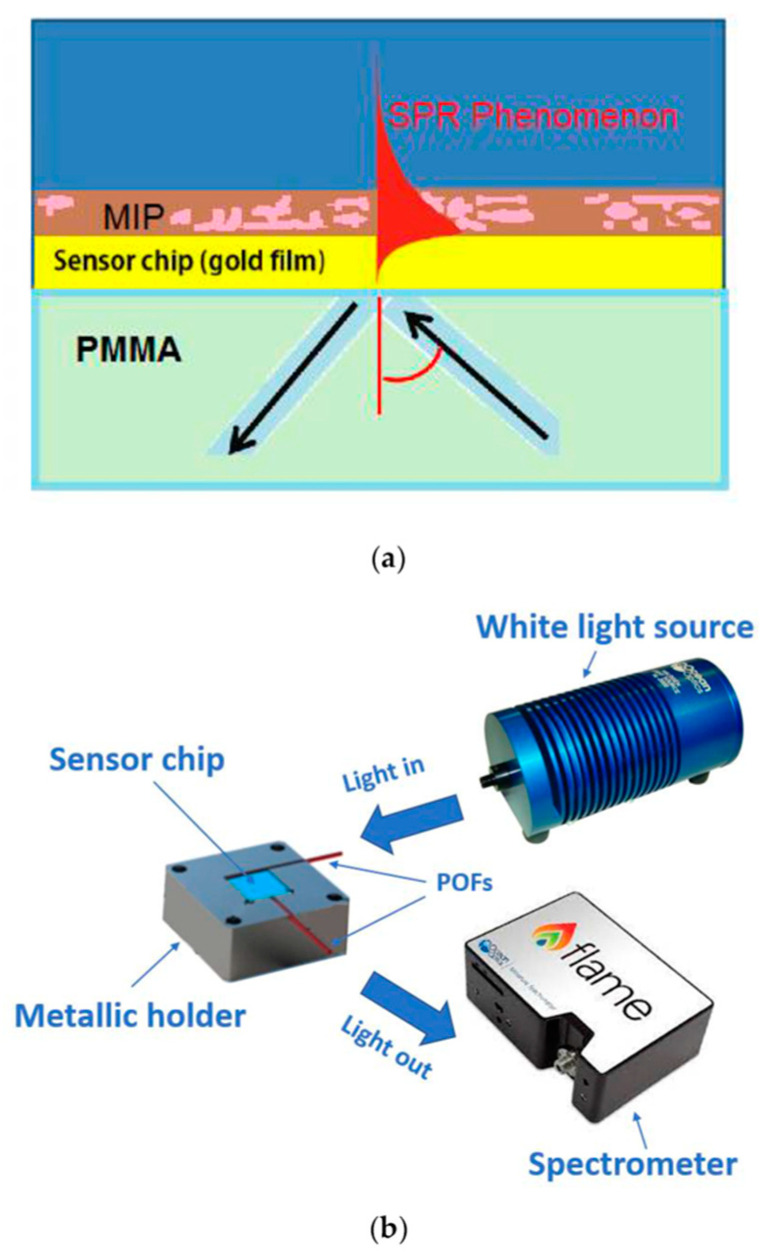
(**a**) The SPR sensor chip’s cross-section. (**b**) Experimental setup. Reprinted from [75] with permission from Cennamo et al.

Recently, Cennamo and co-workers [75] developed a novel SPR probe based on a poly(methyl methacrylate) (PMMA) slab waveguide covered by a gold nanofilm combined with an MIP receptor for the detection of BSA protein. BSA concentrations were tested from 10^−10^ to 10^−5^ M, and the sensor displayed an LOD value of 8.5 × 10^−9^ M. The schematic image of the SPR sensor’s cross-section is depicted in Figure 3.

#### 3.1.2. Myoglobin, Hemoglobin, and Other Proteins

In an earlier study, Osman et al. designed an SPR sensor using the microcontact technique for the detection of myoglobin in human serum [76]. A myoglobin-imprinted poly(hydroxyethylmethacrylate-N-methacryloyl-L-tryptophan methyl ester) [poly(HEMA-MATrp)] nanofilm was prepared on the surface of an SPR sensor. The performance of the MIP-based SPR sensor was evaluated using myoglobin solutions in a buffer and the serum obtained from a patient with acute myocardial infarction. The sensor demonstrated good linearity in the range of 0.1–1.0 μg/mL with an LOD of 87.6 ng/mL.

**Figure 4:**
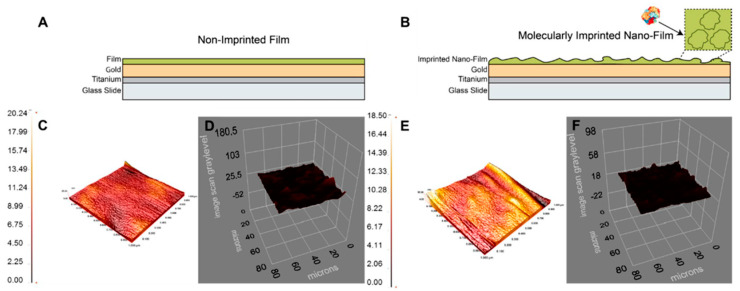
AFM and ellipsometer images of the NIP (**A**,**C**,**D**) and MIP (**B**,**E**,**F**) nanofilms. Reprinted from [77] with permission from Saylan et al.

Saylan and Denizli [77] developed an MIP-based SPR sensor for the detection of hemoglobin. In this study, the template molecule hemoglobin was imprinted on an acrylamide nanofilm using the photopolymerization technique. The MIP nanofilm surface was characterized by AFM and an ellipsometer (Figure 4). The developed SPR sensor showed a linear concentration range between 0.5 µg/mL and 1.0 mg/mL for hemoglobin detection with an LOD value of 0.35 µg/mL.

**Figure 5:**
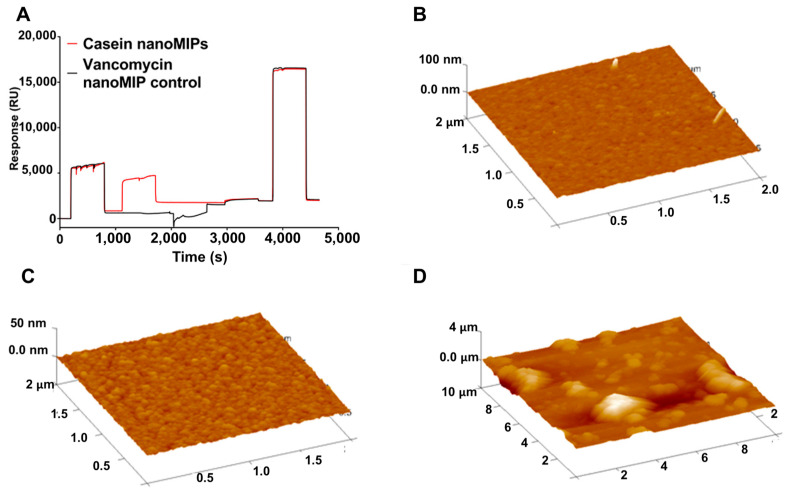
(**A**) SPR sensorgram; AFM image of (**B**) bare gold, (**C**) SAM monolayer, and (**D**) covalently attached nanoMIPs. Reprinted from [78] with permission from Ashley et al.

Casein proteins make up the largest group of proteins in milk and are, therefore, the most common milk protein allergen in food ingredients. Ashley et al. designed MIP nanoparticle (nanoMIPs)-based SPR sensors for the detection of alpha casein protein as an allergen biomarker [78]. The nanoMIPs were synthesized using a solid-phase imprinting method and were subsequently integrated into an SPR sensor for a label-free detection platform. The nanoMIP-modified SPR sensor chip’s surface was characterized using AFM (Figure 5). This MIP-based SPR sensor demonstrated a better performance than existing commercially available ELISA kits, with an LOD of 127 ± 97.6 ng/mL. Notably, this work is very instructive because it illustrates the integration of nanoMIPs with SPR detection very well. 

### 3.2. MIP-Based Piezoelectric Sensors

Piezoelectric sensors are mass-sensitive platforms and have been broadly explored in combination with MIPs. The quartz crystal microbalance (QCM) is the sensor platform commonly used in this field. Previous achievements have demonstrated that MIPs are capable of being integrated in QCM sensors. Furthermore, surface acoustic wave (SAW) sensor applications of MIPs have also been reported in recent years.

#### 3.2.1. Albumin

**Figure 6:**
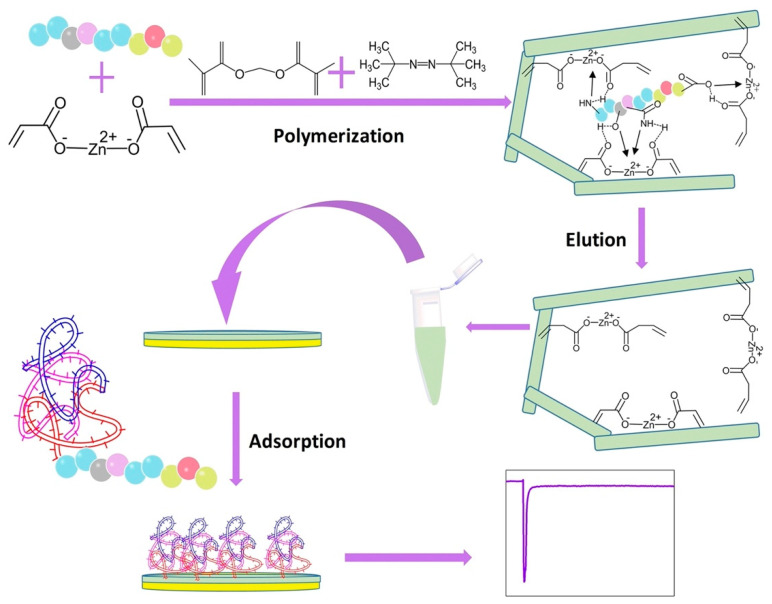
The preparation process of epitope-imprinted-QCM sensor. Reprinted from [79] with permission from Ma et al.

In a successful earlier work, Ma et al. fabricated an epitope-imprinted polymer-based QCM sensor for the detection of albumin [79]. The epitope-imprinted polymer was synthesized in N,N-dimethylformamide using the C-terminus epitope of the template protein HSA and zinc acrylate as the functional monomer. Then, HSA was desorbed from the polymer film to create selective binding sites. Finally, a simple drop-coating technique was employed to obtain the epitope-imprinted QCM sensor chip. According to the results obtained, it was reported that the epitope-MIP was selective for HSA, whereas NIP was not. The *IF* values for horseradish peroxidase (HRP), lysozyme (Lyz), and transferrin (Trf) were calculated to be 2.2, 1.6, and 2.9, respectively, which are all lower than the *IF* of 6.9 for HSA. The fabrication procedure of the epitope-MIP-QCM sensor is shown in Figure 6.

Recently, Sudjarwo et al. reported the use of nanoMIP-coated-QCM-sensing platforms for the detection of albumin using N-Isopropylacrylamide, N-tert-butyl acrylamide, and N, and N′-methylene bisacrylamide as functional monomers and cross-linkers [80]. In this study, Stern–Volmer plots based on fluorescence experiments led to selective binding to HSA with selectivity factors of 1.2 compared to BSA, 1.9 for lysozyme, and 4.1 for pepsin. Subsequently, the binding results were proven by direct QCM assays confirming the binding of nanoMIPs to HSA on QCM surfaces with an LOD value of 80 nM.

#### 3.2.2. Other Proteins

**Figure 7:**
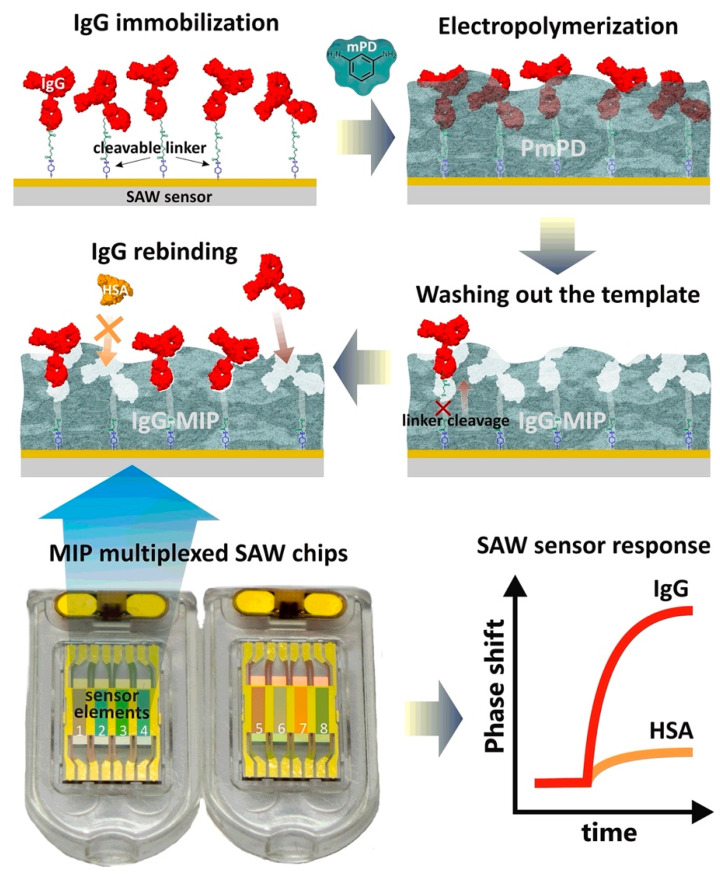
Schematic image of the synthesis procedure for IgG-MIP sensing layer in combination with SAW sensor chip. Reprinted from [81] with permission from Tretjakov et al.

In a seminal work, Tretjakov et al. designed a novel label-free SAW sensor system based on an MIP receptor for the detection of immunoglobulin G (IgG) [81]. The polymeric nanofilms with surface imprints of IgG-MIP were prepared on SAW sensor-multiplexed chips using the electrosynthesis method. The gold surface of the SAW sensor chip was preliminarily cleaned with piranha solution. Then, IgG was immobilized on the cleaned surface through the 3,3′-dithiobis[sulfosuccinimidylpropionate] (DTSSP) cleavable crosslinker (Figure 7) [81]. The results confirmed that polymer film’s thickness affected the recognition performance of IgG-MIP toward IgG, which was optimized by the amount of electrical charge used in the electrodeposition process. The IgG-MIP SAW sensing platform enabled the real-time binding analysis of IgG in the presence of IgA and HSA as the interfering proteins with high sensitivity and selectivity.

**Figure 8:**
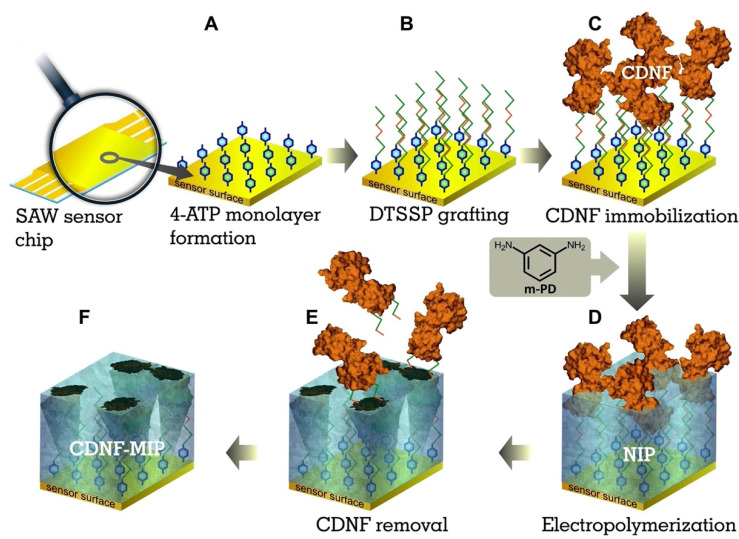
The surface-imprinting technique for synthesis of the CDNF-MIP film on SAW chip’s surface using the electropolymerization method; (**A**) The gold surface of SAW sensor chip and 4-ATP monolayer formation; (**B**) 3,3′-dithiobis [sulfosuccinimidylpropionate] (DTSSP) linker grafting; (**C**) immobilization of the target protein CDNF; (**D**) electropolymerization of poly-mPD; (**E**) removal of target protein CDNF; (**F**) CDNF-MIP layer. Reprinted from [82] with permission from Kidakowa et al.

In another interesting study, Kidakowa and co-workers [82] developed an MIP film-coated SAW sensor to selectively detect the cerebral dopamine neurotrophic factor (CDNF) protein. It is a potential biomarker for the early-stage diagnosis and/or follow-up of neuroprotective therapies. In the first step, they modified the sensor’s surface with the target protein (CDNF) via a 4-ATP/DTSSP linker system with a cleavable S-S bond. Afterwards, the electropolymerization of m-phenylenediamine (mPD) was conducted at a constant potential (0.6 V versus Ag/AgCl/1 M KCl) and the thus formed poly-mPD film was applied to the sensor’s surface (Figure 8). The thicknesses of the poly-mPD films were characterized with an ellipsometer. A CDNF-MIP layer with a 4.7 nm thickness showed the highest relative rebinding toward CDNF.

### 3.3. MIP-Based Electrochemical Sensors

An electrochemical sensor is a device that transforms the interaction of a molecule with a receptor on an electrode’s surface into a measurable analytical signal. Electrochemical sensors use different electroanalytical techniques; the commonly measured characteristics in this regard include amperometry, voltammetry, potentiometry, conductivity, and capacitance or impedance changes [52].

To date, MIPs have been well-integrated in electrochemical sensors as selective recognition layers. In an interesting example of this matching, Duan et al. fabricated a novel MIP-based electrochemical sensor for the highly sensitive detection of BSA [83]. The authors prepared 3D porous electrocatalytic framework materials (AuNPs@NH_2_-MIL-125(Ti) composites) and graphene-modified glassy carbon electrodes. The interaction of the monomer L-Cys with AuNPs through the formation of a SH-Au bond and the electrostatic interaction of BSA via hydrogen bonding were characterized by UV–Vis absorption spectroscopy, and the morphology of the MIP-coated electrode surface was visualized by SEM, TEM, and AFM. The 3D MIP-based electrochemical sensor exhibited a broad linear range of 10^−18^ to 10^−12^ g/mL of BSA under optimal conditions. Notably, an extremely low LOD was found, namely, 4.147 × 10^−19^ g/mL.

**Figure 9:**
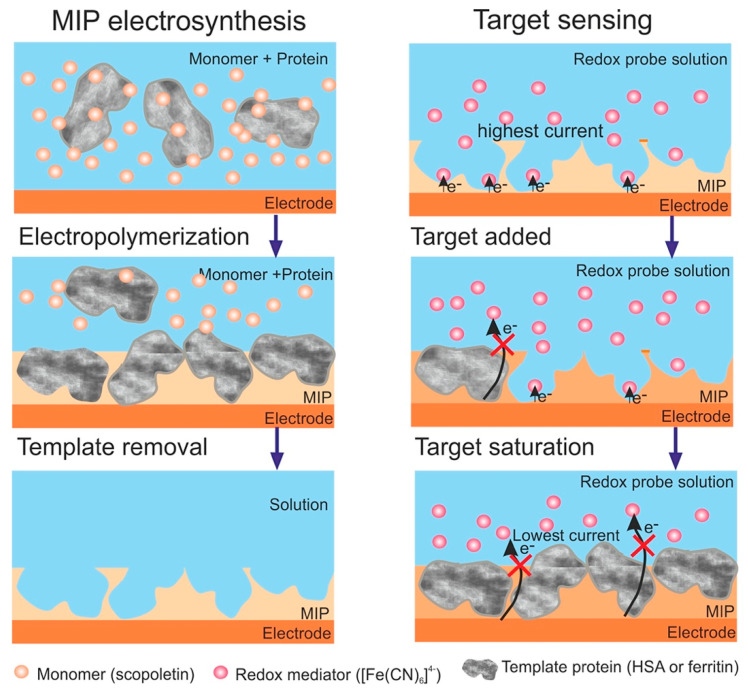
Schematic diagram of the surface-imprinted polyscopoletin nanofilm and its use for protein detection. Reprinted from [84] with permission from Stojanovic et al.

In another successful work, Stojanovic and colleagues [84] developed a polyscopoletin-based MIP nanofilm for the electrochemical detection of elevated HSA in a urine sample. The chemical sensor’s fabrication was achieved by the controlled deposition of MIP nanofilms directly onto an electrode surface. The formation of scopoletin by electropolymerization leads to the formation of an insulating polymer film whose thickness can be adjusted to match the characteristic dimensions of the protein (Figure 9). The developed HSA sensor was successfully used to analyze urine samples of patients with albuminuria. The results suggest that MIP-based sensors may be applicable for measuring high-abundance proteins in a clinical setting.

Cieplak and co-workers [85] designed an electrochemical sensor for the selective and sensitive detection of HSA. They used semi-covalent molecular imprinting for this goal. The HSA-imprinted electrochemical sensor was prepared using a semi-covalent imprinting technique on a Au disk electrode. The MIP thin film was synthesized by oxidative electropolymerization in the presence of a bis(2,2′-bithien-5-yl)methane cross-linker. This MIP was deposited as a thin film on a Au electrode by oxidative potentiodynamic electropolymerization to fabricate an electrochemical sensor. The DPV response of the HSA-imprinted sensor was linear, in the range of 0.8 to 20 µg/mL, with an LOD of 16.6 ng/mL. Moreover, the MIP-based electrochemical sensor’s selectivity against myoglobin, lysozyme, and cytochrome c was reported to be excellent.

In another study, Li et al. developed an MIP electrochemical sensor for the detection of BSA [86]. In this study, the authors proposed a novel method based on enzyme amplification. The detection of BSA was performed using the epitope-imprinting technique. Nonapeptide and o-phenylenediamine were chosen as a template and functional monomer to prepare the MIP thin film. The developed sensor exhibited a linear relationship, with differential pulse voltammetric current variation in the range of 1.0–150 ng/mL, and the LOD was 0.02 ng/mL.

Wang and co-workers [87] developed an MIP-based electrochemical sensor by the electropolymerization of pyrrole in the presence of a template molecule—bovine hemoglobin (BHb). MIP film was coated on an ionic liquid/graphene (IL/GR) modified glassy carbon electrode (IL/GR/GCE). The prepared MIP-based electrochemical sensor had a broad linear range from 1.0 × 10^−7^ to 1.0 mg/L under the optimized conditions with an LOD of 3.09 × 10^−9^ mg/L. In another work, Luo and Liu [88] designed an electrochemical sensor for the selective detection of BHb. They prepared a novel graphene-molecularly imprinted polymer composite. The selective detection of BHb was achieved in the concentration range of 1.0 × 10^−9^ to 1.0 × 10^−1^ mg/mL under optimized experimental conditions and the LOD was 2.0 × 10^−10^ mg/mL.

**Figure 10:**
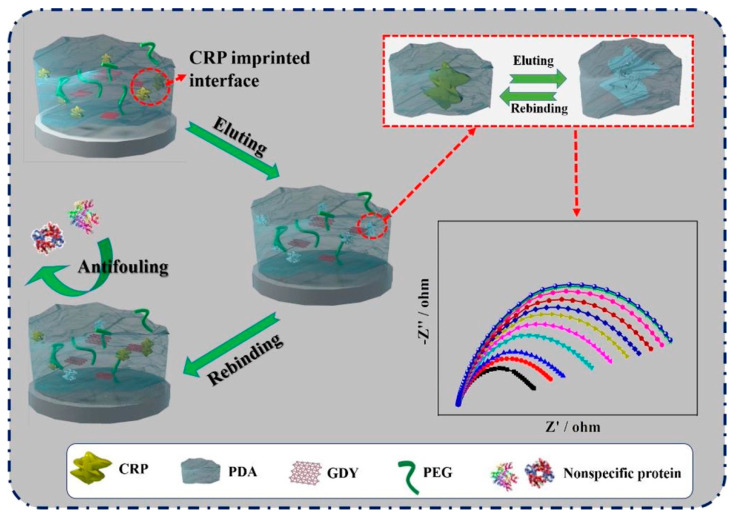
Schematic diagram of the CRP imprinted sensor. Reprinted from [89] with permission from Cui et al.

C-reactive protein (CRP), a very important biomolecule of the immune system, is a clinical biomarker closely associated with cancer and cardiovascular and neurological diseases. Cui et al. prepared a highly sensitive and specific MIP-based CRP electrochemical sensor, in which conductive and biocompatible graphdiyne (GDY) nanosheets and antifouling polyethylene glycol (PEG) were combined to support the recognition of CRP. The designed sensor displayed a broad detection in the range of 10^−5^–10^3^ ng/mL with a 0.41 × 10^−5^ ng/mL LOD value [89]. A schematic diagram of the CRP-imprinted sensor is given in Figure 10.

**Figure 11:**
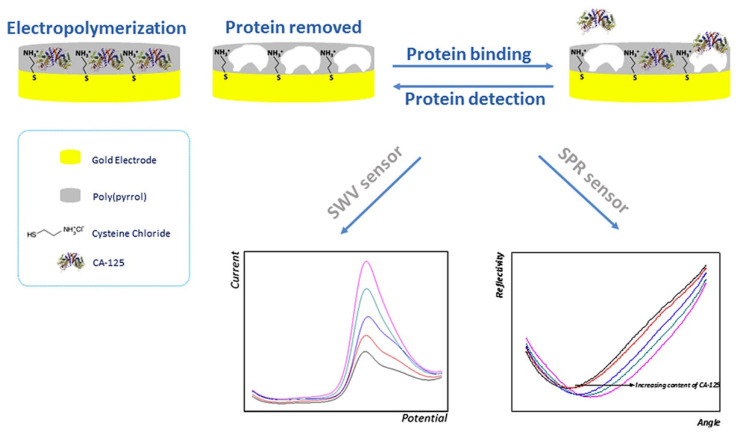
Schematic diagram of the MIP assembly for electrochemical and optical sensors. Reprinted from [90] with permission from Rebelo et al.

Rebelo and co-workers [90] developed an electrochemical sensor based on MIPs for the detection of the CA-125 biomarker. In this study, carbohydrate antigen 125 (CA-125) protein, an ovarian cancer biomarker, was chosen as the target for the production of an MIP-based sensor. The determination of the CA-125 biomarker was made through the comparison of two transducers: an electrochemical (square wave voltammetry-SWW) and an optical (SPR) transducer (Figure 11). The electrochemical sensor presented good analytical performance, yielding remarkable selectivity and an LOD of 0.01 U/mL. This sensor provided a linear concentration range from 0.01 U/mL to 500 U/mL.

Optical, piezoelectric, and electrochemical sensing platforms based on molecularly imprinted polymers have special relevance in real-life applications and point-of-care testing in biological fluids. A summary of MIP-based sensors developed for the detection of template proteins is given in Table 1. The detection of proteins in complex biological samples has far-reaching importance across proteomics and molecular medicine. MIP-based sensors possess a unique combination of features, such as robustness, high affinity, specificity, and cost-effective preparation, which makes them attractive alternatives to natural receptors. Thus, to date, MIPs have been employed for the direct detection of various proteins.

## 4. Conclusions

Currently, the label-free and real-time sensing of proteins is in high demand not only in clinical practice but also in fundamental research as an alternative to the commonly employed label-based detection methodologies, which can affect the interfacial activity of the resulting protein and are very laborious.

High-selectivity molecules or coatings, such as antibodies or enzymes, are of great importance in biology, chemistry, and diagnostics. Manufacturing these natural receptors, however, is expensive or difficult. They are also biomolecules, which limits their lifetime and applicability. Molecular imprinting is a technology designed to overcome these challenges. MIPs constitute a specific type of polymer that is formed according to the volume, shape, and molecular structure of the template. MIPs have many advantages, such as their tailor-made nature, which enables the recognition of a broad range of target analytes or compounds; excellent chemical and physical stability; compatibility with organic media; potential reusability; simple engineering; and cost-effectiveness. Moreover, MIPs have highly selective affinity for target molecules employed in the imprinting process, their synthesis is relatively cheap, and their longer degradation times provide the recognition sites to retain for quite a long time at room temperature. Occasionally, cost is also an extra factor; in this regard, MIPs are usually cheap when compared to the cost of natural antibodies and are relatively cost-effective materials considering their long-term storage properties and reusable features. Furthermore, the chemical stability and thermal stability of MIPs are very good. Therefore, these benefits have garnered significant interest in MIPs. MIP-based sensors are systems that synergistically combine the advantages of MIPs, particularly their selectivity, with the intrinsic gains of sensors. Sensors offer simplicity, sensitivity, portability, and fast analysis in a user-friendly way. Consequently, these properties found their way into MIPs, which are such vital elements in the health monitoring field. The general strategy lies in sending a measurable signal to the user. To achieve this result, an MIP is attached to the surface of a transducer, which detects binding events between the MIP and the target analyte, thus sending the aforementioned signal. This process usually necessitates the preparation of an MIP in film format. At that point, sensitivity of the device depends on the thickness and porosity of the film, which need to be optimized.

In this context, developing mechanically robust and susceptible sensors for detecting proteins of interest could help diagnose and monitor various illnesses. Their rapid production, cost-effectiveness, simple handling, and portability are the main factors that have resulted in these sensors’ wide investigation in healthcare monitoring. This is where the molecular-imprinting technique comes into play. The selective and sensitive response of a sensor via a precisely designed biorecognition element is achievable through MIPs. Their high stability and resistance to degradation, low cost, and simple production techniques make them ideal candidates for this task. These materials are cheaper to synthesize and can be produced in large quantities with good reproducibility. However, some weaknesses of molecular imprinting technology need to be overcome, such as template leakage, poor accessibility to binding sites, low binding capacity, and non-specific binding. The epitope-imprinting technique is among the most promising approaches for the formation of efficient synthetic adsorbents for proteins and, simultaneously, avoids the limitations involved in the manipulation of all biological receptors. The surface-imprinting technique is currently one of the most popular and general methods for protein imprinting and successfully solves the diffusion limitation problem caused by the large sizes of proteins.

Recently, the producers of affinity-based sensing devices started to base their development strategies on MIPs thanks to their advantages such as low cost, good reproducibility, and good stability compared to natural bioreceptors. Among the plethora of biomimetic recognition schemes utilizing supramolecular approaches, MIPs have proven their potential as synthetic receptors in numerous application analyses and sensor technologies. It is reasonable to predict that commercial applications of MIPs for sensors will emerge in the foreseeable future, given the considerable number of reports of MIP-based protein sensing in the literature. However, there is still a great deal of research to be performed, especially with respect to in vivo applications and the market production of MIP-based protein sensors.

## Figures and Tables

**Table 1:** MIP-based sensors developed for the detection of protein targets.

Sensor Type	Template Protein	Cross Linker/Functional Monomer	Ref.
**Optical**	Albumin (HSA, BSA)	Ethylene glycol dimethacrylate/N-methacryloyl-L-leucine methyl ester	[73]
		(3-aminophenyl)boronic acid (3-APBA)	[74]
		poly(methyl methacrylate) (PMMA) slab waveguide	[75]
	Myoglobin	hydroxyethylmethacrylate/N-methacryloyl-L-tryptophan methyl ester	[76]
	Hemoglobin	Methylenebisacrylamide/acrylamide	[77]
	Casein	N-tert-butylacrylamide (TBA), N-(3-aminopropyl)methacrylamide HCl (APM)/N,N′-methylenebis(acrylamide) (BIS)	[78]
**Piezoelectric**	Albumin (HSA, BSA)	N,N-dimethylformamide/zinc acrylate	[79]
		N-Isopropylacrylamide, N-tert-butyl acrylamide/N, N′-methylene bisacrylamide	[80]
	Immunoglobulin G (IgG)	3,3′dithiobis[sulfosuccinimidylpropionate] (DTSSP)	[81]
	Cerebral dopamine neurotrophic factor (CDNF)	m-phenylenediamine (mPD)/4- 4-aminothiophenol (4-ATP)-3,3′-dithiobis [sulfosuccinimidylpropionate] (DTSSP)	[82]
**Electrochemical**	Albumin (HSA, BSA)	amino-functionalized Ti-benzenedicarboxylate porous metal-organic frameworks (Au/NH2-MIL-125(Ti))/l-cysteine (L-Cys)	[83]
		N, N′-methylene bisacrylamide/N-tert-butyl acrylamide	[84]
		5,5′,5′-methanetriyltris(2,2′-bithiophene)/2,2′-bithiophene-5-carboxylic acid 1, p-bis(2,2′-bithien-5-yl)methylalanine	[85]
		o-phenylenediamine	[86]
	Hemoglobin	glassy carbon electrode (GCE)	[87]
		Graphite oxide (GO)/dopamine	[88]
	C-reactive protein (CRP)	biocompatible graphdiyne (GDY) nanosheets/anti-fouling polyethylene glycol (PEG)	[89]
	Carbohydrate antigen 125 (CA-125) protein	Poly(pyrrol)/Cysteine chloride	[90]

## Data Availability

Not applicable.

## References

[1] Erdőssy J., Horváth V., Yarman A., Scheller F.W., Gyurcsányi R.E. (2016). Electrosynthesized Molecularly Imprinted Polymers for Protein Recognition. TrAC Trends Anal. Chem..

[2] Bedwell T.S., Whitcombe M.J. (2016). Analytical Applications of MIPs in Diagnostic Assays: Future Perspectives. Anal. Bioanal. Chem..

[3] Turner N.W., Jeans C.W., Brain K.R., Allender C.J., Hlady V., Britt D.W. (2006). From 3D to 2D: A Review of the Molecular Imprinting of Proteins. Biotechnol. Prog..

[4] Xing R., Wen Y., Dong Y., Wang Y., Zhang Q., Liu Z. (2019). Dual Molecularly Imprinted Polymer-Based Plasmonic Immunosandwich Assay for the Specific and Sensitive Detection of Protein Biomarkers. Anal. Chem..

[5] Kryscio D.R., Fleming M.Q., Peppas N.A. (2012). Protein Conformational Studies for Macromolecularly Imprinted Polymers. Macromol. Biosci..

[6] Han Z., Xu Y., Tian H., Liang J., Sun D. (2021). Enhanced Ammonia Adsorption and Separation by a Molecularly Imprinted Polymer after Acid Hydrolysis of Its Ester Crosslinker. J. Hazard. Mater..

[7] Devkota L., Nguyen L.T., Vu T.T., Piro B. (2018). Electrochemical Determination of Tetracycline Using AuNP-Coated Molecularly Imprinted Overoxidized Polypyrrole Sensing Interface. Electrochim. Acta.

[8] Pacheco J.G., Silva M.S.V., Freitas M., Nouws H.P.A., Delerue-Matos C. (2018). Molecularly Imprinted Electrochemical Sensor for the Point-of-Care Detection of a Breast Cancer Biomarker (CA 15-3). Sens. Actuators B Chem..

[9] Parisi O.I., Ruffo M., Malivindi R., Vattimo A.F., Pezzi V., Puoci F. (2020). Molecularly Imprinted Polymers (MIPs) as Theranostic Systems for Sunitinib Controlled Release and Self-Monitoring in Cancer Therapy. Pharmaceutics.

[10] El-Schich Z., Abdullah M., Shinde S., Dizeyi N., Rosén A., Sellergren B., Wingren A.G. (2016). Different Expression Levels of Glycans on Leukemic Cells—a Novel Screening Method with Molecularly Imprinted Polymers (MIP) Targeting Sialic Acid. Tumor Biol..

[11] Nerantzaki M., Michel A., Petit L., Garnier M., Ménager C., Griffete N. (2022). Biotinylated Magnetic Molecularly Imprinted Polymer Nanoparticles for Cancer Cell Targeting and Controlled Drug Delivery. Chem. Commun..

[12] Demir B., Lemberger M.M., Panagiotopoulou M., Medina Rangel P.X., Timur S., Hirsch T., Tse Sum Bui B., Wegener J., Haupt K. (2018). Tracking Hyaluronan: Molecularly Imprinted Polymer Coated Carbon Dots for Cancer Cell Targeting and Imaging. ACS Appl. Mater. Interfaces.

[13] Chen Z., Liu X., Huang C., Li J., Shen X. (2020). Artificial Cytochrome c Mimics: Graphene Oxide-Fe(III) Complex-Coated Molecularly Imprinted Colloidosomes for Selective Photoreduction of Highly Toxic Pollutants. ACS Appl. Mater. Interfaces.

[14] Haupt K., Mosbach K. (2000). Molecularly Imprinted Polymers and Their Use in Biomimetic Sensors. Chem. Rev..

[15] Spivak D.A. (2005). Optimization, Evaluation, and Characterization of Molecularly Imprinted Polymers. Adv. Drug Deliv. Rev..

[16] Polyakov M.V. (1931). Adsorption Properties and Structure of Silica Gel. Zhur Fiz Khim.

[17] Wulff G. (2002). Enzyme-like Catalysis by Molecularly Imprinted Polymers. Chem. Rev..

[18] Withers G.S. (1993). Drug Assay Using Antibody Mimics. Nature.

[19] Wulff G. (1972). The Use of Polymers with Enzyme-Analogous Structures for the Resolution of Racemates. Angrew. Chem. Internat. Ed..

[20] Saylan Y., Akgönüllü S., Yavuz H., Ünal S., Denizli A. (2019). Molecularly Imprinted Polymer Based Sensors for Medical Applications. Sensors.

[21] Arshady R., Mosbach K. (1981). Synthesis of Substrate-selective Polymers by Host-guest Polymerization. Die Makromol. Chemie Macromol. Chem. Phys..

[22] Wulff G., Sarhan A. (1972). Macromolecular Colloquium. Angew. Chemie Int. Ed. Eng..

[23] Chiappini A., Pasquardini L., Bossi A.M. (2020). Molecular Imprinted Polymers Coupled to Photonic Structures in Biosensors: The State of Art. Sensors.

[24] Wei X., Samadi A., Husson S.M. (2005). Synthesis and Characterization of Molecularly Imprinted Polymers for Chromatographic Separations. Sep. Sci. Technol..

[25] Topçu A.A., Kılıç S., Özgür E., Türkmen D., Denizli A. (2022). Inspirations of Biomimetic Affinity Ligands: A Review. ACS Omega.

[26] Ahmad A., Nantasenamat C., Piacham T. (2017). Molecularly Imprinted Polymer for Human Viral Pathogen Detection. Mater. Sci. Eng. C.

[27] Jahanban-Esfahlan A., Roufegarinejad L., Jahanban-Esfahlan R., Tabibiazar M., Amarowicz R. (2020). Latest Developments in the Detection and Separation of Bovine Serum Albumin Using Molecularly Imprinted Polymers. Talanta.

[28] Ndunda E.N. (2020). Molecularly Imprinted Polymers—A Closer Look at the Control Polymer Used in Determining the Imprinting Effect: A Mini Review. J. Mol. Recognit..

[29] Dinc M., Esen C., Mizaikoff B. (2019). Recent Advances on Core–Shell Magnetic Molecularly Imprinted Polymers for Biomacromolecules. TrAC-Trends Anal. Chem..

[30] Chen L., Wang X., Lu W., Wu X., Li J. (2016). Molecular Imprinting: Perspectives and Applications. Chem. Soc. Rev..

[31] Mujahid A., Iqbal N., Afzal A. (2013). Bioimprinting Strategies: From Soft Lithography to Biomimetic Sensors and Beyond. Biotechnol. Adv..

[32] Zahedi P., Ziaee M., Abdouss M., Farazin A., Mizaikoff B. (2016). Biomacromolecule Template-Based Molecularly Imprinted Polymers with an Emphasis on Their Synthesis Strategies: A Review. Polym. Adv. Technol..

[33] Drzazgowska J., Schmid B., Su R.D., Altintas Z. (2020). Self-Assembled Monolayer Epitope Bridges for Molecular Imprinting and Cancer Biomarker Sensing. Anal. Chem..

[34] Rossetti C., Świtni M.A., Halvorsen T.G., Cormack P.A.G., Sellergren B., Reubsaet L. (2017). Automated Protein Biomarker Analysis: On-Line Extraction of Clinical Samples by Molecularly Imprinted Polymers. Sci. Rep..

[35] Haupt K., Medina Rangel P.X., Bui B.T.S. (2020). Molecularly Imprinted Polymers: Antibody Mimics for Bioimaging and Therapy. Chem. Rev..

[36] BelBruno J.J. (2019). Molecularly Imprinted Polymers. Chem. Rev..

[37] Chen M., Zhong M., Johnson J.A. (2016). Light-Controlled Radical Polymerization: Mechanisms, Methods, and Applications. Chem. Rev..

[38] Poma A., Turner A.P.F., Piletsky S.A. (2010). Advances in the Manufacture of MIP Nanoparticles. Trends Biotechnol..

[39] Wackerlig J., Lieberzeit P.A. (2015). Molecularly Imprinted Polymer Nanoparticles in Chemical Sensing – Synthesis, Characterisation and Application. Sens. Actuators B Chem..

[40] Singh M., Singh S., Singh S.P., Patel S.S. (2020). Recent Advancement of Carbon Nanomaterials Engrained Molecular Imprinted Polymer for Environmental Matrix. Trends Environ. Anal. Chem..

[41] Schirhagl R. (2014). Bioapplications for Molecularly Imprinted Polymers. Anal. Chem..

[42] Akgönüllü S., Denizli A. (2023). Molecular Imprinting-Based Sensors: Lab-on-Chip Integration and Biomedical Applications. J. Pharm. Biomed. Anal..

[43] Mahony J.O., Nolan K., Smyth M.R., Mizaikoff B. (2005). Molecularly Imprinted Polymers - Potential and Challenges in Analytical Chemistry. Anal. Chim. Acta.

[44] Wang X., Chen G., Zhang P., Jia Q. (2021). Advances in Epitope Molecularly Imprinted Polymers for Protein Detection: A Review. Anal. Methods.

[45] Boonsriwong W., Chunta S., Thepsimanon N., Singsanan S., Lieberzeit P.A. (2021). Thin Film Plastic Antibody-Based Microplate Assay for Human Serum Albumin Determination. Polymers.

[46] Surapong N., Burakham R. (2021). Magnetic Molecularly Imprinted Polymer for the Selective Enrichment of Glyphosate, Glufosinate, and Aminomethylphosphonic Acid Prior to High-Performance Liquid Chromatography. ACS Omega.

[47] Akgönüllü S., Yavuz H., Denizli A. (2021). Development of Gold Nanoparticles Decorated Molecularly Imprinted–Based Plasmonic Sensor for the Detection of Aflatoxin M1 in Milk Samples. Chemosensors.

[48] Akgönüllü S., Yavuz H., Denizli A. (2020). SPR Nanosensor Based on Molecularly Imprinted Polymer Film with Gold Nanoparticles for Sensitive Detection of Aflatoxin B1. Talanta.

[49] Haupt K., Linares A.V., Bompart M., Bui B.T.S., Haupt K. (2012). Molecularly Imprinted Polymers. Molecular Imprinting.

[50] Mostafiz B., Arjomand S., Banan K., Afsharara H., Hatamabadi D., Mousavi P., Mustansar C., Keçili R., Ghorbani-Bidkorbeh F. (2021). Molecularly Imprinted Polymer-Carbon Paste Electrode (MIP-CPE)-Based Sensors for the Sensitive Detection of Organic and Inorganic Environmental Pollutants: A Review. Trends Environ. Anal. Chem..

[51] Lu H., Xu S. (2022). Carbon Nanofibers Coated with Fe_3_O_4_ Nanoparticles and MnO_2_ Nanosheets Further Modified with Molecularly Imprinted Polydopamine for Fluorescence Sensing of Carcinoembryonic Antigen. ACS Appl. Nano Mater..

[52] Ahmad O.S., Bedwell T.S., Esen C., Garcia-Cruz A., Piletsky S.A. (2019). Molecularly Imprinted Polymers in Electrochemical and Optical Sensors. Trends Biotechnol..

[53] Beluomini M.A., da Silva J.L., de Sá A.C., Buffon E., Pereira T.C., Stradiotto N.R. (2019). Electrochemical Sensors Based on Molecularly Imprinted Polymer on Nanostructured Carbon Materials: A Review. J. Electroanal. Chem..

[54] Naresh V., Lee N. (2021). A Review on Biosensors and Recent Development of Nanostructured Materials-Enabled Biosensors. Sensors.

[55] Yáñez-Sedeño P., Campuzano S., Pingarrón J.M. (2017). Electrochemical Sensors Based on Magnetic Molecularly Imprinted Polymers: A Review. Anal. Chim. Acta.

[56] Naseri M., Mohammadniaei M., Sun Y., Ashley J. (2020). The Use of Aptamers and Molecularly Imprinted Polymers in Biosensors for Environmental Monitoring: A Tale of Two Receptors. Chemosensors.

[57] Akhoundian M., Rüter A., Shinde S. (2017). Ultratrace Detection of Histamine Using a Molecularly-Imprinted Polymer-Based Voltammetric Sensor. Sensors.

[58] Holthoff E.L., Stratis-cullum D.N., Hankus M.E. (2011). A Nanosensor for TNT Detection Based on Molecularly Imprinted Polymers and Surface Enhanced Raman Scattering. Sensors.

[59] De Middeleer G., Dubruel P., De Saeger S. (2016). Characterization of MIP and MIP Functionalized Surfaces: Current State-of-the-Art. TrAC Trends Anal. Chem..

[60] Boysen R.I. (2019). Advances in the Development of Molecularly Imprinted Polymers for the Separation and Analysis of Proteins with Liquid Chromatography. J. Sep. Sci..

[61] Martín-Esteban A. (2022). Green Molecularly Imprinted Polymers for Sustainable Sample Preparation. J. Sep. Sci..

[62] Ratautaite V., Samukaite-Bubniene U., Plausinaitis D., Boguzaite R., Balciunas D., Ramanaviciene A., Neunert G., Ramanavicius A. (2021). Molecular Imprinting Technology for Determination of Uric Acid. Int. J. Mol. Sci..

[63] He J.X., Pan H.Y., Xu L., Tang R.Y. (2021). Application of Molecularly Imprinted Polymers for the Separation and Detection of Aflatoxin. J. Chem. Res..

[64] Akgönüllü S., Denizli A. (2021). Piezoelectric biosensors for virus detection. Biosensors for Virus Detection.

[65] Peeters M., Kobben S., Jiménez-Monroy K.L., Modesto L., Kraus M., Vandenryt T., Gaulke A., Van Grinsven B., Ingebrandt S., Junkers T. (2014). Thermal Detection of Histamine with a Graphene Oxide Based Molecularly Imprinted Polymer Platform Prepared by Reversible Addition-Fragmentation Chain Transfer Polymerization. Sens. Actuators B Chem..

[66] Sharma P.S., Pietrzyk-Le A., D’Souza F., Kutner W. (2012). Electrochemically Synthesized Polymers in Molecular Imprinting for Chemical Sensing. Anal. Bioanal. Chem..

[67] Lakshmi D., Bossi A., Whitcombe M.J., Chianella I., Fowler S.A., Subrahmanyam S., Piletska E.V., Piletsky S.A. (2009). Electrochemical Sensor for Catechol and Dopamine Based on a Catalytic Molecularly Imprinted Polymer-Conducting Polymer Hybrid Recognition Element. Anal. Chem..

[68] Esen C., Czulak J., Cowen T., Piletska E., Piletsky S.A. (2019). Highly Efficient Abiotic Assay Formats for Methyl Parathion: Molecularly Imprinted Polymer Nanoparticle Assay as an Alternative to Enzyme-Linked Immunosorbent Assay. Anal. Chem..

[69] Rico-Yuste A., Carrasco S. (2019). Molecularly Imprinted Polymer-Based Hybrid Materials for the Development of Optical Sensors. Polymers.

[70] Esen C., Piletsky S.A. (2021). Surface Plasmon Resonance Sensors Based on Molecularly Imprinted Polymers. Plasmonic Sensors and their Applications.

[71] Valerio C., Theocharidou E., Davenport A., Agarwal B. (2016). Human Albumin Solution for Patients with Cirrhosis and Acute on Chronic Liver Failure: Beyond Simple Volume Expansion. World J. Hepatol..

[72] Shajari D., Bahari A., Gill P. (2018). Fast and Simple Detection of Bovine Serum Albumin Concentration by Studying Its Interaction with Gold Nanorods. Colloids Surf. A Physicochem. Eng. Asp..

[73] Esentürk M.K., Akgönüllü S., Yılmaz F., Denizli A. (2019). Molecularly Imprinted Based Surface Plasmon Resonance Nanosensors for Microalbumin Detection. J. Biomater. Sci. Polym. Ed..

[74] Wang Y., Wei T.X. (2013). Surface Plasmon Resonance Sensor Chips for the Recognition of Bovine Serum Albumin via Electropolymerized Molecularly Imprinted Polymers. Chinese Chem. Lett..

[75] Arcadio F., Zeni L., Perri C., D’Agostino G., Chiaretti G., Porto G., Minardo A., Cennamo N. (2021). Bovine Serum Albumin Protein Detection by a Removable SPR Chip Combined with a Specific MIP Receptor. Chemosensors.

[76] Osman B., Uzun L., Beşirli N., Denizli A. (2013). Microcontact Imprinted Surface Plasmon Resonance Sensor for Myoglobin Detection. Mater. Sci. Eng. C.

[77] Saylan Y., Denizli A. (2018). Molecular Fingerprints of Hemoglobin on a Nanofilm Chip. Sensors.

[78] Ashley J., Shukor Y., D’Aurelio R., Trinh L., Rodgers T.L., Temblay J., Pleasants M., Tothill I.E. (2018). Synthesis of Molecularly Imprinted Polymer Nanoparticles for α-Casein Detection Using Surface Plasmon Resonance as a Milk Allergen Sensor. ACS Sens..

[79] Ma X., He X., Li W., Zhang Y. (2017). Epitope Molecularly Imprinted Polymer Coated Quartz Crystal Microbalance Sensor for the Determination of Human Serum Albumin. Sensors Actuators B Chem..

[80] Sudjarwo W.A.A., Dobler M.T., Lieberzeit P.A. (2022). QCM-Based Assay Designs for Human Serum Albumin. Anal. Bioanal. Chem..

[81] Tretjakov A., Syritski V., Reut J., Boroznjak R., Öpik A. (2016). Molecularly Imprinted Polymer Film Interfaced with Surface Acoustic Wave Technology as a Sensing Platform for Label-Free Protein Detection. Anal. Chim. Acta.

[82] Kidakova A., Boroznjak R., Reut J., Öpik A., Saarma M., Syritski V. (2020). Molecularly Imprinted Polymer-Based SAW Sensor for Label-Free Detection of Cerebral Dopamine Neurotrophic Factor Protein. Sens. Actuators B Chem..

[83] Duan D., Yang H., Ding Y., Ye D., Li L., Ma G. (2018). Three-Dimensional Molecularly Imprinted Electrochemical Sensor Based on Au NPs@Ti-Based Metal-Organic Frameworks for Ultra-Trace Detection of Bovine Serum Albumin. Electrochim. Acta.

[84] Stojanovic Z., Erdőssy J., Keltai K., Scheller F.W., Gyurcsányi R.E. (2017). Electrosynthesized Molecularly Imprinted Polyscopoletin Nanofilms for Human Serum Albumin Detection. Anal. Chim. Acta.

[85] Cieplak M., Szwabinska K., Sosnowska M., Chandra B.K.C., Borowicz P., Noworyta K., D’Souza F., Kutner W. (2015). Selective Electrochemical Sensing of Human Serum Albumin by Semi-Covalent Molecular Imprinting. Biosens. Bioelectron..

[86] Li M.-X., Wang X.-H., Zhang L.-M., Wei X.-P. (2017). A High Sensitive Epitope Imprinted Electrochemical Sensor for Bovine Serum Albumin Based on Enzyme Amplifying. Anal. Biochem..

[87] Wang Z., Li F., Xia J., Xia L., Zhang F., Bi S., Shi G., Xia Y., Liu J., Li Y. (2014). An Ionic Liquid-Modified Graphene Based Molecular Imprinting Electrochemical Sensor for Sensitive Detection of Bovine Hemoglobin. Biosens. Bioelectron..

[88] Luo J., Jiang S., Liu X. (2014). Electrochemical Sensor for Bovine Hemoglobin Based on a Novel Graphene-Molecular Imprinted Polymers Composite as Recognition Element. Sens. Actuators B Chem..

[89] Cui M., Che Z., Gong Y., Li T., Hu W., Wang S. (2022). A Graphdiyne-Based Protein Molecularly Imprinted Biosensor for Highly Sensitive Human C-Reactive Protein Detection in Human Serum. Chem. Eng. J..

[90] Rebelo T., Costa R., Brandão A., Silva A.F., Sales M.G.F., Pereira C.M. (2019). Molecularly Imprinted Polymer SPE Sensor for Analysis of CA-125 on Serum. Anal. Chim. Acta.

